# Supplementary material for: Rapid and robust endoscopic content area estimation: A lean GPU-based pipeline and curated benchmark dataset
Source: Comput Methods Biomech Biomed Eng Imaging Vis. Author manuscript; Available in PMC 2024 Apr 10. (PMC7615255; doi:10.1080/21681163.2022.2156393)
Supplement: Appendix [file EMS177365-supplement-Appendix.pdf]

Appendix A. Parameters

Table A1. A tabulation of the parameters for our content area estimation methods. The Parameters are presented in two blocks. The upper block are the main parameters of the methods and may be of interest to end users whishing to tune, although the default values should be sufficient in most use cases. The second block of parameters are provided for completeness but are likely of little interest to end users.

Parameter	Value	Method	Description
N	16	both	The number of image strips to investigate.
t_g	20	handcrafted	Gradient magnitude threshold for edge point scoring.
t_θ	30	handcrafted	Gradient direction threshold for edge point scoring.
t_ι	25	handcrafted	Preceding maximum intensity threshold for edge point scoring.
t_{ps}	0.03	both	Point score threshold to filter low scoring points.
t_{cs}	0.06	both	Final circle score threshold to filter out low scoring circles.
α	8	both	Determines to what extent strips are weighted to the vertical extremes of the image.
t_{px}	3	handcrafted	Candidate edge points within this many pixels of the border are discarded.
t_{ri}	3	both	The distance in pixels within which a candidate edge point is counted as an inlier for the RANSAC circle fitting
r_{min}	0.1	both	Minimum radius of a found circle (multiple of image width).
r_{max}	0.8	both	Maximum radius of a found circle (multiple of image width).
d_{max}	0.2	both	Maximum distance between the center of the image and a found circle (multiple of image width).
